# Maternal obesity (Class I-III), gestational weight gain and maternal leptin levels during and after pregnancy: a prospective cohort study

**DOI:** 10.1186/s40608-016-0108-2

**Published:** 2016-05-20

**Authors:** Sara Carlhäll, Marie Bladh, Jan Brynhildsen, Ing-Marie Claesson, Ann Josefsson, Gunilla Sydsjö, Annika Thorsell, Marie Blomberg

**Affiliations:** Department of Obstetrics and Gynaecology and Department of Clinical and Experimental Medicine, Linköping University, 58185 Linköping, Sweden; Division of Cell Biology, Department of Clinical and Experimental Medicine, Faculty of Health Sciences, Linköping University, 58185 Linköping, Sweden

**Keywords:** Maternal obesity, Leptin, Gestational weight gain, Pregnancy and body mass index

## Abstract

**Background:**

Maternal obesity is accompanied by maternal and fetal complications during and after pregnancy. The risks seem to increase with degree of obesity. Leptin has been suggested to play a role in the development of obesity related complications. Whether maternal leptin levels differ between obese and morbidly obese women, during and after pregnancy, have to our knowledge not been previously described. Neither has the association between maternal leptin levels and gestational weight gain in obese women. The aim was to evaluate if maternal plasma leptin levels were associated with different degrees of maternal obesity and gestational weight gain.

**Methods:**

Prospective cohort study including women categorized as obesity class I-III (*n* = 343) and divided into three gestational weight gain groups (*n* = 304). Maternal plasma leptin was measured at gestational week 15, 29 and 10 weeks postpartum. Maternal Body Mass Index (BMI) was calculated from early pregnancy weight. Gestational weight gain was calculated using maternal weight in delivery week minus early pregnancy weight. The mean value and confidence interval of plasma-leptin were analysed with a two-way ANOVA model. Interaction effect between BMI and gestational weight gain group was tested with a two-way ANOVA model.

**Results:**

The mean maternal leptin concentrations were significantly higher in women with obesity class III compared to women in obesity class I, at all times when plasma leptin were measured. The mean leptin concentrations were also significantly higher in women with obesity class II compared to women in obesity class I, except in gestational week 29. There was no difference in mean levels of plasma leptin between the gestational weight gain groups. No significant interaction between BMI and gestational weight gain group was found.

**Conclusions:**

Plasma leptin levels during and after pregnancy were associated with obesity class but not with degree of gestational weight gain. These results are in concordance with epidemiological findings where the risk of obstetric complications increases with increased maternal obesity class. The effect on obstetric outcome by degree of gestational weight gain is less pronounced than the adverse effects associated with maternal obesity.

## Background

Maternal obesity is accompanied by several complications for both mother and infant during and after pregnancy [1, 2]. Maternal risks are gestational diabetes mellitus (GDM), pre-eclampsia (PE), hypertension, increased duration of labour, caesarean delivery, postpartum haemorrhage etc. [1, 3–7]. Fetal risks include neural tube and heart defects, macrosomia, stillbirth etc. [1, 3, 8]. The risks for a majority of obesity related complications during pregnancy, for both mother and infant, seem to increase with increasing degree of obesity [1, 3, 8]. A pronounced gestational weight gain (GWG) influences pregnancy outcome as well, but to a lesser extent than maternal obesity [3, 9].

The pathogenetic mechanisms of obesity on obstetric outcome have not yet been clarified. Obesity induces inflammatory processes, associated with some pregnancy outcomes like PE, gestational hypertension and GDM [10, 11]. Leptin, secreted by adipose tissue, is one of several adipokines. It is believed to act as a pro-inflammatory cytokine that might have a role in development of obesity related complications [12–14]. Serum leptin concentrations correlate with body mass index (BMI) and percentage of body fat in humans [15, 16]. During pregnancy, leptin is also produced by the placenta and plays an important role for normal fetal development and growth [12, 13, 17, 18]. High serum leptin levels seem to be associated with adverse pregnancy outcomes, such as PE, GDM and macrosomia [12, 17, 19, 20].

Prior studies have indicated an association between leptin, body fat mass or maternal BMI, during pregnancy [21–23]. Misra et al. demonstrated that leptin concentrations in overweight/obese women were higher but increased at a significantly lower rate across gestation compared to normal-weight women [24]. A similar leptin pattern across gestation, which differed according to pre-pregnancy maternal BMI, was observed in a smaller Brazilian cohort [25]. These studies mainly focused on the longitudinal trends in maternal leptin levels during pregnancy and included few obese and morbidly obese women [22, 24–26]. Hendler et al. demonstrated that maternal leptin levels in the third trimester, increased with pre-pregnancy BMI in 20 obese women [19]. Walsh et al. compared leptin levels in a cohort of women subdivided into those who exceeded the American Institute of Medicine’s (IOM) GWG guidelines and those with recommended GWG. They found no difference in leptin levels in early pregnancy but maternal leptin concentration in gestational week 28 were higher in women who exceeded the GWG recommendation [27].

Whether maternal plasma leptin levels differ substantially between obese and morbidly obese women, during and after pregnancy, have to our knowledge not been previously described. There are no available data on the association between maternal leptin levels in obese women, during and after pregnancy, and recommended GWG, based on the IOM guidelines [28]. Accordingly, the aim of the present study was to estimate whether maternal plasma leptin levels were associated with different degrees of maternal obesity (obesity class I-III) during and after pregnancy and further to evaluate maternal plasma leptin levels during and after pregnancy in obese women with different levels of GWG, based on IOM guidelines.

## Methods

**Fig. 1:**
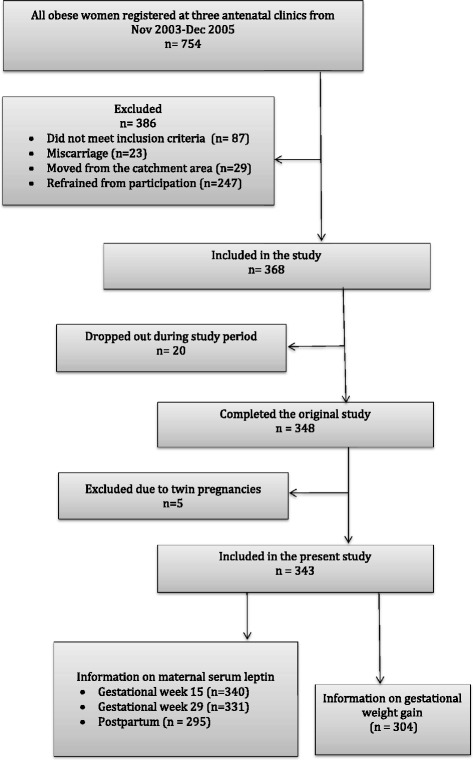
Flow-chart of the cohort

Data were collected as part of a prospective cohort study on obese pregnant women [29]. Between November 2003 and December 2005, all obese pregnant women (*n* = 754), consecutively registered in early pregnancy in three antenatal clinics in Sweden, were approached to the study [29]. The inclusion criteria were early pregnancy BMI ≥ 30 and knowledge of the Swedish language. The exclusion criteria were pre-pregnant diagnosis of diabetes mellitus, thyroid dysfunction or psychiatric disease treated with neuroleptic drugs. A flow chart of the study is presented in Fig. 1. Out of the 615 women who were eligible and invited to participate, 368 women were included in the study and 348 completed the study. Additionally five women, who expected twins, were excluded, leaving 343 women with singleton pregnancies as the present study population.

The study-population was categorized in three obesity classes of early pregnancy BMI, based on the WHO definition; class I obesity = BMI 30–34.9, class II obesity = BMI 35–39.9 (morbid obesity) and class III obesity = BMI ≥ 40 (morbid obesity) [30]. BMI was defined as body weight in kilograms divided by height in meter squared (kg/m^2^). All women had their weight and height recorded in the gestation week eight to ten, which enabled calculation of BMI.

The study-population was also divided into three groups of GWG: < 5 kg, 5–9 kg and > 9 kg. In 2009 the IOM published guidelines advising optimal weight gain during pregnancy, based on pre-pregnancy BMI. According to these guidelines, the recommended GWG for obese women is 5–9 kg [28]. To calculate GWG we used maternal weight in delivery week minus early pregnancy weight. If maternal weight in delivery week was missing, the weight 1 or 2 weeks before delivery, was used. A total number of 304 women had information on GWG. Gestational age was calculated based on fetal biometry at the first trimester ultrasound. Data on maternal characteristics and pregnancy complications were retrieved from computerized patient records.

Maternal plasma leptin levels were measured in week 15 and 29 of gestation and 10 weeks postpartum. The women fasted prior to sampling of maternal blood. Maternal blood was collected in the morning in all patients.

For leptin analyses blood was collected in a test tube with a clot activator and gel for serum separation. One hour after sampling, the blood was centrifuged, aliquoted and serum was stored at −70° Celsius until analyses.

The maternal plasma leptin concentration was measured using a direct sandwich based ELISA (Millipore, Billerica, USA). Human leptin was captured by a polyclonal antibody on a 96-well microtiter plate and unbound material washed away. A secondary monoclonal biotinylated antibody was added to the captured human leptin complex followed by streptavidin-horseradish peroxidase. The enzyme activity was measured spectrophotometrically (Victor 3, PerkinElmer, Waltham, MA, USA) at 450 nm after a last step of acidification of the sample products. Increased absorbance was directly proportional to the amount of captured human leptin in unknown samples derived from a generated standard-curve. In a sample size of 25 μL, the limit of sensitivity of the assay was 0.2 ng +/− 2 SD. The within and between assay variation was 4.6 and 6.2 % respectively. The specificity of the assay was 100 % for human leptin. No cross-reactivity was found for human pro-insulin, insulin, insulin-growth factor I and II or Glucagon.

Differences in categorical demographic variables as well as obstetric outcomes, between maternal obesity class I-III, were analysed using Pearson’s Chi-square test and ANOVA was used for analysing differences in continuous variables and outcomes (maternal age, birth weight, GWG and gestational length in weeks.) Categorical variables and outcomes were expressed as numbers (percentage) and if continuous as mean (SD).

The mean value and confidence interval of plasma-leptin during and after pregnancy in women with obesity class I-III and in obese women in three different GWG groups were analysed with a two-way ANOVA model. (Bonferroni adjusted within each gestational week).

Interaction effect between Maternal BMI and GWG was tested with a two-way ANOVA model.

General linear models were used to test possible confounding in addition to the main effect model of BMI and gestational weight gain.

All analyses were performed using IBM SPSS version 23 (IBM Inc, Armonk, NY). A *p*-value <0.05 was considered statistically significant.

The Regional Ethical Review Board in Linköping, Sweden approved this study (No 03–231).

## Results

Out of the 343 women included in the study, 223 (65.0 %) were classified as obesity class I, 77 (22.4 %) were defined as obesity class II and 43 (12.5 %) as obesity class III. Of these 343 women, 304 (88.6 %) women had information that enabled calculation of gestational weight gain. A total number of 50 (16.4 %) women gained less than 5 kg (low GWG), 87 (28.6 %) gained 5–9 kg (recommended GWG) and 167 (54.9 %) exceeded the IOM gestational weight gain guidelines, i.e. more than 9 kg.

Maternal demographic characteristics, pregnancy outcome and complications over the BMI strata are presented in Tables 1 and 2. No differences in mean maternal age, parity, mean gestational age, birth weight, smoking and alcohol usage in first trimester were found between the groups. Mean GWG, was significant lower in obesity class III compared to obesity class I and class II. The distribution of GDM and hypertension before pregnancy did not differ substantially between the obesity classes. The prevalence of PE increased with increasing BMI.

**Table 1:** Maternal characteristics and pregnancy outcome in relation to maternal obesity class. Continuous variables Mean (Std)

	Obesity class I	Obesity class II	Obesity class III	*P*-value
	*N* = 223	*N* = 77	*N* = 43	
Maternal age (years)	30.0 (4.7)	30.4 (4.4)	29.0 (5.3)	0.322
Gestational length (full weeks)	39.2 (2.0)	39.8 (1.4)	39.0 (2.6)	0.076
Birth-weight (gram)	3708 (594)	3713 (448)	3670 (811)	0.920
Weight gain during pregnancy (kg)	10.6 (5.6)	9.6 (6.0)	7.7 (6.1)	0.015
	*N* = 203	*N* = 66	*N* = 35	

*P* < 0.05 statistical significance in difference between obesity class I-III groups

**Table 2:** Maternal characteristics and pregnancy outcome in relation to maternal obesity class. Categorical variables *N* (%)

	Obesity class I	Obesity class II	Obesity class III	*P*-value
Parity				0.714
Nulliparous	96 (43.0)	36 (46.8)	21 (48.8)	
Multiparous	127 (57.0)	41 (53.2)	22 (51.2)	
Smoking in pregnancy				0.059
No	206 (93.2)	72 (94.7)	35 (83.3)	
Yes	15 (6.8)	4 (5.3)	7 (16.7)	
Alcohol in first trimester				#
No/rarely	223 (100.0)	77 (100.0)	43 (100.0)	
Yes	0 (0.0)	0 (0.0)	0 (0.0)	
Pre-term (<37 weeks)				0.046
No	209 (94.6)	76 (100.0)	38 (90.5)	
Yes	12 (5.4)	0 (0.0)	4 (9.5)	
Post-term (≥42 weeks)				0.993
No	206 (93.2)	71 (93.4)	39 (92.9)	
Yes	15 (6.8)	5 (6.6)	3 (7.1)	
GDM				0.505
No	211 (94.6)	75 (97.4)	40 (93.0)	
Yes	12 (5.4)	2 (2.6)	3 (7.0)	
Pre-eclampsia				0.009
No	211 (94.6)	68 (88.3)	35 (81.4)	
Yes	12 (5.4)	9 (11.7)	8 (18.6)	
Hypertension pre-pregnancy				#
No	221 (99.1)	77 (100.0)	42 (97.7)	
Yes	2 (0.9)	0 (0.0)	1 (2.3)	

GDM = Gestational diabetes mellitus

*P* < 0.05 statistical significance in difference between obesity class I-III groups

# = Assumption for chi^2^-test not fulfilled

In gestational week 15, samples from 340 (99.1 %) women were available for measurement of leptin levels and in gestational week 29, 331 (96.5 %) women. At 10 weeks post partum, leptin levels were measured in 295 (86.0 %) women (Fig. 1).

The median week, 25th and 75th percentiles and the lowest and highest value of pregnancy week or week postpartum when blood was drawn for leptin analyses, are presented in Table 3. There was no significant correlation between gestational week, when blood was drawn for leptin analyses, and the value of leptin, within each time-period of leptin measurement (week 15 *p* = 0.33, week 29 *p* = 0.45 and 10 weeks postpartum *p* = 0.70). Since there was no significant correlation between gestational week at leptin analyses and the value of leptin, within each time-period of leptin measurement, the leptin analyses have not been adjusted for gestational week at time of leptin measurement.

**Table 3:** Gestational week and weeks postpartum at leptin measurements

	Gestational week at measurement 1	Gestational week at measurement 2	Weeks postpartum at measurement 3
	*N* = 340	*N* = 331	*N* = 295
Min	10	24	6
25 percentile	14	28	9
Median	15	29	10
75 percentile	16	30	12
Max	22	33	28

No significant interaction was found between maternal BMI and GWG, therefore the analyses on leptin and GWG-group were not adjusted for BMI and vice versa.

**Fig. 2:**
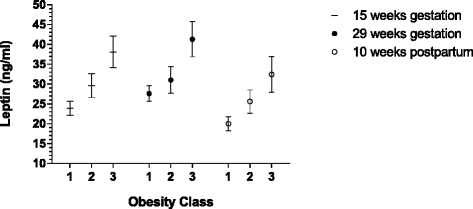
Plasma leptin values during and after pregnancy in women with obesity class I-III (in figure class 1–3). Leptin values are mean and 95 % confidence interval (Bonferroni adjusted within each gestational week). Non-overlapping confidence interval implies significant differences (*p* < 0.05)

The mean maternal plasma leptin concentrations and 95 % confidence interval in the three obesity classes are presented in Fig. 2. Mean maternal leptin concentrations during and after pregnancy seem to be associated with degree of maternal obesity. The mean maternal leptin concentrations were significantly higher in women with obesity class III compared to women in obesity class I, at all times when plasma leptin were measured. The mean leptin concentrations were also significantly higher in women with obesity class II compared to women in obesity class I, except in gestational week 29. Postpartum the mean leptin concentrations were higher with increasing obesity category, although not reaching statistically significant difference between women in obesity class II and III.

**Fig. 3:**
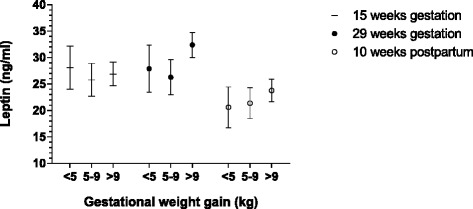
Plasma leptin values during and after pregnancy in obese women divided in low, recommended and excessive gestational weight gain groups. Leptin values are mean and 95 % confidence interval (Bonferroni adjusted within each gestational week). Non-overlapping confidence interval implies significant differences (*p* < 0.05)

The mean maternal plasma leptin concentrations and 95 % confidence interval, in the GWG groups are presented in Fig. 3. Mean maternal leptin concentrations during and after pregnancy do not seem to be associated with degree of GWG. There was no significant difference in plasma-leptin levels during and after pregnancy in obese women classified according to degree of GWG, with one exception. Obese women with > 9 kg GWG had significantly higher mean plasma leptin concentration in pregnancy week 29 compared to obese women with recommended GWG (5–9 kg).

No significant confounding was found using general linear models. The resulting model included only the main effects of BMI and GWG (data not shown).

## Discussion

This large study included 223 women in obesity class I and 120 women with morbid obesity (obesity class II and III) and showed higher leptin levels during and after pregnancy in women with morbid obesity, compared to women in obesity class I. Thus, at each leptin measurement during and after pregnancy maternal leptin levels were significantly higher among women in obesity class III compared to obesity class I women. There was no significant difference in maternal leptin levels at 15 weeks gestation and postpartum between low, recommended and excessive GWG. At 29 weeks gestation measurement women with excessive weight gain had a significantly higher leptin levels than women in obesity class II.

It is well described in epidemiological studies that the risk-estimates of adverse obstetric and neonatal outcome vary substantially between obesity class I and obesity class III [8, 31]. Leptin and other adipokines have been studied in relation to different adverse outcomes as well and have been found to be elevated in complicated pregnancies [12, 17, 19, 20]. Increased leptin levels have been found in women with PE [19, 20]. If such proteins are involved in the well described step-wise increased risk of adverse outcomes for both the mother and the infant for each higher obesity class, the levels of the adipokines should vary substantially with the degree of obesity. Results in this study add knowledge on this issue by showing a significant difference in leptin levels between the three degrees of obesity.

There are some previous studies indicating that maternal leptin levels are associated with maternal pre-pregnancy BMI [19, 22, 24–26]. Misra et al. studied the longitudinal effects of maternal pre-pregnancy BMI on serum leptin concentrations during pregnancy in 143 women, categorized as non-overweight or overweight/obese. Leptin levels were analysed five times during pregnancy. Leptin concentrations were higher in the overweight/obese group but increased at a significantly lower rate across gestation, compared to normal weight [24]. Similar results were presented in Brazilian study including 42 women, categorized as normal-weight or overweight/obese, according to their pre-pregnancy BMI [25] Yang et al. reported that serum leptin concentrations in 114 women, with a normal singleton pregnancy, correlated with gestational age, maternal bodyweight and BMI in the three trimesters of pregnancy [22]. This is in accordance with findings in the present study. However there are differences. The majority of previous studies were based on relatively small cohorts and often analysed obese and overweight women as one group or with few obese individuals included.

This study showed that there was no difference in maternal plasma leptin levels during and after pregnancy in obese women with excessive GWG, compared to obese women with recommended GWG, with one exception. Among women with excessive weight gain in gestational week 29, women in obesity class III had significantly higher leptin levels compared to those in obesity class II. Although the confidence limits are close to each other but not overlapping. It is possible that excessive weight gain in late second trimester contributes to significantly higher leptin levels. It seemed though that the degree of weight gain during pregnancy in obese women is of minor importance for the actual serum leptin value during and after pregnancy compared to early pregnancy BMI. Walsh et al. prospectively studied serum leptin concentration in 621 pregnant women, subdivided into those who did (*n* = 267) and those who did not (*n* = 354) exceed the IOM gestational weight gain guidelines, regardless of BMI in early pregnancy. Maternal plasma leptin concentrations were measured in early pregnancy and in gestational week 28. The leptin levels were significantly higher in women with excessive weight gain in gestational week 28 but not in early pregnancy [27].

Excessive GWG has previously been shown to increase risk factors related to obstetric and neonatal outcome in obese women [9], although to a lower magnitude than risks associated with morbidly obesity. Epidemiological data have shown that the most favourable obstetric and neonatal outcome among obese women is related to low GWG, except for birth of small for gestational age infants [32, 33].

This study has certain strengths and limitations. One of the strength is the large number of obese individuals included, which enabled analyses of three subgroups of obesity. In addition, BMI was based on measured weight in week eight to ten of gestation. The IOM guidelines are based on pre-pregnancy weight. However during the first two months of pregnancy, the weight gain is minimal [34]. Further, information on GWG was available in a majority of the obese women included, which enabled a division into three groups according to degree of GWG. A thorough baseline evaluation on maternal comorbidity and demographic variables and continuous registration of maternal complications in all study subjects were also available. This information made it possible to demonstrate that there were no major differences between the different obesity classes concerning variables that could have influenced the maternal leptin levels.

There are limitations of this study. The postpartum measurement of leptin could only be performed in 86 % of the study subjects since not all women attended the proposed postpartum control. Apart from this, almost all study subjects (98.5 %) had complete information on leptin concentration during pregnancy. It must also be kept in mind when interpreting the data, that although a large sample size in the study-population, numbers in certain subgroups were low. Another limitation is the lack of data on maternal leptin at time of delivery.

## Conclusions

In conclusion, this study demonstrated that maternal plasma leptin levels during and after pregnancy differed significantly between the women with obesity and morbid obesity. Further no major differences in leptin levels were defined between women with low, recommended and excessive GWG. That is in concordance with observations done in epidemiological studies where the most severe complications during and after pregnancy occur among morbidly obese women and the amount of GWG could alter these risks marginally.

### Ethics approval and consent to participate

The Regional Ethical Review Board in Linköping, Sweden approved this study. (No 03–231.) Written informed consent was received from all study participants.

### Consent for publication

Not appliciable.

### Availability of data and materials

Data supporting our findings can be sent upon request.

## Abbreviations

BMI: body mass index
GWG: gestational weight gain
GDM: gestational diabetes mellitus
PE: pre-eclampsia
IOM: Institute of Medicine
WHO: World Health Organization.
